# Understanding the mechanisms of TAVI durability through computational modelling: a multidisciplinary review

**DOI:** 10.1093/ehjdh/ztag020

**Published:** 2026-02-03

**Authors:** Elisa Rauseo, Laura Bevis, Xu Chen, Steffen E Petersen, Anthony Mathur, Gregory G Slabaugh, Caroline H Roney

**Affiliations:** William Harvey Research Institute, NIHR Barts Biomedical Research Centre, Queen Mary University of London, Charterhouse Square, London EC1M 6BQ, UK; Barts Heart Centre, Barts Health NHS Trust, W. Smithfield, London EC1A 7BE, UK; Digital Environment Research Institute, Queen Mary University of London, 67-75 New Rd, London E1 1HH, UK; Digital Environment Research Institute, Queen Mary University of London, 67-75 New Rd, London E1 1HH, UK; School of Engineering and Materials Science, Queen Mary University of London, Mile End Road, London E1 4NS, UK; Digital Environment Research Institute, Queen Mary University of London, 67-75 New Rd, London E1 1HH, UK; Department of Medicine, University of Cambridge, Cambridge, UK; William Harvey Research Institute, NIHR Barts Biomedical Research Centre, Queen Mary University of London, Charterhouse Square, London EC1M 6BQ, UK; Barts Heart Centre, Barts Health NHS Trust, W. Smithfield, London EC1A 7BE, UK; NIHR Barts Biomedical Research Centre, Queen Mary University of London, Charterhouse Square, London, UK; Barts Heart Centre, Barts Health NHS Trust, W. Smithfield, London EC1A 7BE, UK; NIHR Barts Biomedical Research Centre, Queen Mary University of London, Charterhouse Square, London, UK; Centre for Cardiovascular Medicine and Devices, William Harvey Research Institute, Queen Mary University of London, London, UK; Digital Environment Research Institute, Queen Mary University of London, 67-75 New Rd, London E1 1HH, UK; Digital Environment Research Institute, Queen Mary University of London, 67-75 New Rd, London E1 1HH, UK; School of Engineering and Materials Science, Queen Mary University of London, Mile End Road, London E1 4NS, UK

**Keywords:** Computational modelling, Simulations, Transcatheter aortic valve implantation, Durability, Structural valve degeneration, Thrombosis

## Abstract

As transcatheter aortic valve implantation (TAVI) expands to younger populations, durability has become a concern, requiring a lifetime rather than a single-procedure perspective. While clinical trials suggest comparable mid-term performance to surgical bioprostheses, data beyond 10 years remain limited, particularly for bicuspid valves, valve-in-valve procedures, and complex anatomies. Computational modelling combines patient anatomy and device design in computer-based simulations to study valve performance under physiological loading. Applied to TAVI, these models can reproduce implantation, evaluate mechanical stresses, and simulate blood flow, providing mechanistic insights into deterioration processes, including altered leaflet loading, stent deformation, and thrombosis-prone flow. Although these simulations do not directly assess durability, they use surrogate metrics linked with these mechanisms, helping identify factors that may influence longevity and guide design and procedural refinements. Clinically, modelling could support patient-specific planning and reintervention strategies, informing decisions across the valve-replacement pathway, an important consideration as younger patients are likely to undergo multiple lifetime procedures. Integrating these tools into pre-procedural planning may help anticipate challenges such as coronary access, annular geometry, and redo feasibility. However, current studies report elements of verification and field-level validation, but none complete a pre-specified, calibrated surrogate-to-outcome validation with uncertainty/sensitivity analysis; thus, durability predictions remain exploratory. Progress needs transparent verification, field checks vs. bench or imaging, surrogate calibration to data, outcome testing in independent cohorts, and routine uncertainty/sensitivity reporting, with close clinician-engineer collaboration. This review underscores the need for a multidisciplinary approach and provides a critical analysis of the available tools and their potential to advance long-term outcomes.

## Graphical Abstract

**Graphical Abstract:**
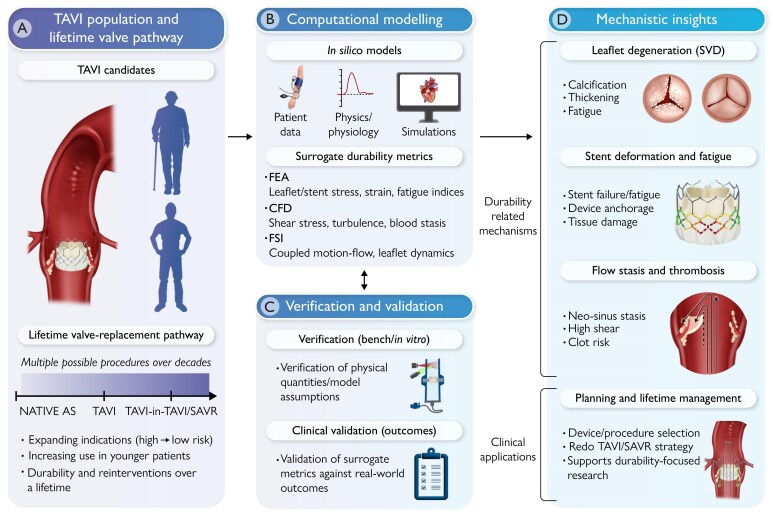
Understanding the mechanisms of TAVI durability through computational modelling: a multidisciplinary review. Expanding indications for transcatheter aortic valve implantation (TAVI) from high- to low-risk patients, including younger individuals, makes durability and lifetime valve-replacement pathways increasingly important (Panel A). Computational modelling (Panel B) uses anatomical and clinical data together with finite element analysis (FEA), computational fluid dynamics (CFD), and fluid–structure interaction (FSI) to generate surrogate durability metrics (stress, strain, shear, stasis), which require verification and validation through *in vitro* testing and clinical outcome data (Panel C). These models provide mechanistic insight into durability-related mechanisms, and may ultimately support device/procedure selection, redo TAVI/SAVR strategies, and durability-focused research for lifetime management (Panel D). AS, aortic stenosis; SAVR, surgical aortic valve replacement; SVD, structural valve degeneration.

## Introduction

Transcatheter aortic valve replacement (TAVI) is a well-established treatment for severe aortic stenosis (AS).^1,2^ Initially reserved for inoperable or high-risk patients as an alternative to surgical aortic valve replacement (SAVR), its use has expanded to intermediate and low-risk individuals.^3–5^ With younger patients increasingly receiving TAVI, long-term device durability has become central to supporting its use over SAVR.

Like surgical bioprostheses, transcatheter aortic valves (TAVs) deteriorate over time, raising concerns about reintervention, particularly in younger patients.^3,6^ TAVI also introduces anatomical and procedural factors that may accelerate this process, and create specific failure mechanisms. The main limitation to durability is structural valve degeneration (SVD), characterized by irreversible leaflet damage from calcification or thickening.^7,8^ Leaflet thrombosis, reported more frequently in TAVI than SAVR, may further accelerate SVD.^7,8^ These mechanisms are multifactorial, interrelated, and remain incompletely understood, underscoring the need for research specific to TAVI. Such investigations are constrained by the limited long-term data available. Most durability evidence comes from older, high-risk cohorts, and early-generation devices, leaving younger and low-risk patients underrepresented.^3,5,9^ Consequently, durability and degeneration mechanisms in these groups remain uncertain and will take years to characterize through clinical follow-up.

Computational modelling offers a complementary way to investigate TAV performance and durability mechanisms beyond clinical or experimental studies. By combining patient anatomy and device design in computer-based simulations, these models can reproduce implantation, quantify leaflet and stent stresses, and simulate blood flow under physiological conditions. Applied to TAVI, modelling helps explore mechanical and haemodynamic factors linked to degeneration, such as altered leaflet loading, stent deformation, and thrombosis-prone flow that are difficult to capture *in vivo*. Although not a replacement for long-term clinical data, modelling can offer early mechanistic insights and surrogate metrics that correlate with deterioration processes. These can help inform device optimization, procedural planning, and patient selection, especially for younger patients who are likely to undergo multiple interventions during their lifetime.

Several engineering and clinical studies have applied computational modelling to TAVI, mainly to simulate implantation and predict procedural complications. Fewer have examined valve degeneration mechanisms, and those often rely on surrogate metrics that only partially represent long-term processes. Previous reviews offered broad overviews of modelling applications in TAVI, but they have generally adopted a technical perspective aimed at bioengineering audiences, with limited emphasis on the potential clinical interpretation of the metrics used for TAV-specific degeneration mechanisms.^10–12^ As a result, the translational potential of computational modelling to clarify durability-related mechanisms and inform clinical decision-making remains underexplored.

This review provides a systematic, multidisciplinary evaluation of computational modelling approaches used to investigate TAVI durability mechanisms, focusing on SVD and thrombosis, two key, but poorly understood processes. The literature was identified, screened, and appraised collaboratively by clinical and engineering researchers to ensure technical accuracy and clinical relevance. This review evaluates how current modelling can enhance understanding of durability-related mechanisms, identify methodological and validation gaps, and highlight their potential role in supporting long-term TAVI management and future device optimization.

The paper begins with an overview of TAV degeneration mechanisms and computational modelling approaches, introduces common metrics for assessing TAVI durability, and then presents the findings of the systematic review, with a discussion of clinical implications, limitations, and future directions.

### Mechanisms of TAV degeneration

Bioprosthetic valve degeneration is a complex, progressive phenomenon involving biological, pathological, and haemodynamic factors affecting TAV performance and durability. It includes two mechanisms: SVD, the main cause, and non-SVD.^13,14^

SVD refers to irreversible leaflet or frame changes that impair TAV function, presenting as regurgitation and/or stenosis.^13–15^ Structural deterioration may include leaflet tears, thickening, calcification, abnormal leaflet mobility, or strut/frame fracture.^14,15^ These changes typically arise beyond five years, posing a key concern for long-term durability.

Non-SVD includes early post-procedural issues unrelated to intrinsic valve degeneration but capable of accelerating SVD by altering haemodynamics and mechanical stresses on TAV devices.^13,14,16^ These include paravalvular leak (PVL) due to poor sealing, prosthesis malposition, and patient-prosthesis mismatch (PPM), where an undersized prosthesis limits cardiac output. Other non-SVD mechanisms are TAV leaflet thrombosis and endocarditis, as these processes are reversible and do not directly involve permanent leaflet change. However, persistent thrombosis can cause irreversible damage and progression to SVD.^13,14,16^ SVD and non-SVD are thus interconnected, making it complex to determine the sequence of events leading to valve deterioration.

Factors specific to TAVI can also impact device durability, including device type and procedural aspects (e.g. valve crimping, balloon expansion, asymmetric expansion, residual native annular calcification).^16^

Diagnosing SVD remains challenging due to overlapping symptoms and imaging findings with other complications, such as PPM.^13–15^ Subclinical leaflet thrombosis (SLT) is also hard to detect, even with multi-detector computed tomography (MDCT).^14,15^ These limitations hinder accurate durability data collection in TAVI and complicate comparisons across clinical trials. Standardized SVD definitions based on leaflet morphology and haemodynamic criteria have been introduced to improve consistency in reporting.^15^ Meanwhile, computational modelling offers a valuable complement to improve diagnostics, clarify mechanisms, and help anticipate durability outcomes in clinical care.

### Computational modelling in TAVI

Computational modelling can provide detailed, non-invasive insights into patient physiology and clinical procedures, particularly when conventional techniques are impractical or high-risk. By integrating numerical methods with medical imaging and clinical data, *in silico* models simulate physiological scenarios and procedures, potentially supporting clinical decision-making.

While TAVI durability is complex to investigate directly, computational modelling can assess a range of metrics known to be associated with or promote valve degeneration, serving as surrogates measures of durability. For instance, by analysing the magnitude and distribution of mechanical stresses, blood stasis and wall shear stress, models can provide information on factors such as the likelihood and high-risk regions for mechanical device failure and TAV thrombosis (*Table 1*). Combined with clinical investigations, this approach can improve understanding of degeneration pathways and likely durability outcomes.

**Table 1:** Computational modelling methods in TAVI

Method	Main use	Application for TAVI	Main benefits	Requirements	Limitations	Useful metrics
FEA	Structural behaviour	Stent and/or leaflet fatigue/failure, effects of implantation site, device crimping, under-/over-expansion	Understand failure/fatigue mechanisms, identify problematic factors or regions of TAVI components, improve planning and design for TAVI durability	TAVI geometry and material properties (stent and/or leaflets), patient anatomy (inc. calcifications)	Fluid motion neglected, studies may neglect leaflets and skirts, model stability difficult for some procedural steps (e.g. crimping)	Mechanical stresses within devices, stresses at implantation site causing vascular trauma
CFD	Fluid behaviour	Assess aortic flow through and around TAVI device, hydrodynamic performance, thrombosis, fluid stresses and turbulent flow features affecting device durability	Understand effect of flow on stresses and stress distributions within TAVI devices, identify problematic flow patterns indicative of mechanical damage/stress and potential thrombosis	TAVI geometry for scenario of interest (e.g. implanted configuration), patient anatomy (inc. calcifications), fluid properties (e.g. density, viscosity), flow information at inflow/outflow boundaries	Does not account for boundary movement, valve often considered in open or closed state, sensitive to conditions imposed at inflows/outflows, studies may use idealized geometries for implanted stents and leaflets	Flow turbulence, shear stresses imposed on devices, flow stasis/stagnation, flow recirculation, shear stresses and accumulated shear stress on platelets, shear stress causing vascular trauma
FSI	Interacting structural and fluid behaviours	Detailed insights into flow-TAVI interactions, leaflet function, durability and thrombosis, valve performance	Detailed insights into complex flow-leaflet interactions, insights into leaflet motion and resultant stresses, degeneration, thrombus formation, and overall durability	TAVI geometry and material properties (stent and leaflets), patient anatomy and tissue properties, fluid properties (blood density, viscosity)	Required material properties often difficult to obtain or must be modelled/approximated, models may account for deformation of some structures but not others, e.g. leaflet motion with rigid aortic wall, more computationally demanding than FEA and CFD	Mechanical stresses within devices, flow turbulence, shear stresses imposed on devices, flow stasis/stagnation, flow recirculation, shear stresses and accumulated shear stress on platelets, shear stress causing vascular trauma

CFD, Computational fluid dynamics; FEA, Finite element analysis; FSI, fluid–structure interaction; TAVI, Transcatheter Aortic Valve Implantation.

Three main methods are used to study TAVI durability: Finite Element Analysis (FEA), Computational Fluid Dynamics (CFD), and Fluid–Structure Interaction (FSI). Their detailed descriptions are available elsewhere; here we outline the key points relevant to this review and summarizes potential applications and metrics in *Table 1*.^17–19^ FEA is primarily used for structural analyses, while CFD is used to study fluid-only problems with rigid boundaries to the flow. Coupling them in an FSI approach models more complex fluid–structure interactions, providing a more comprehensive view; however, this comes at increased computational cost. These methods can complement *in vitro* experiments and clinical observations to investigate long-term TAVI performance and optimize device durability.

Computational approaches can vary in their degree of patient-specificity. Both generalized *in silico* models, based on known physiological or population values, and patient-specific models, incorporating individual data, offer value in TAVI. Generic or idealized models can help identify key parameters and biophysical mechanisms affecting durability, while patient-specific models, tailored to anatomical and clinical data, offer more accurate predictions, particularly for underrepresented groups that may differ from population averages. The further consideration of *in silico* simulations of *in vitro* flow experiments and regulatory tests can help validate numerical methods and accelerate TAVI design development. Large virtual patient cohorts, generated from patient-specific models, can be used to test device designs in *in silico* clinical trials, to optimize durability and performance under physiological conditions.^20^

Accurate modelling requires appropriate clinical data, assumptions, numerical methods, and material properties to capture patient-specific interactions and valve mechanics. The complexity of the task and the range of available methods necessitate a multidisciplinary approach.

## Methods

### Search strategy and selection criteria

A systematic literature search was conducted across five databases (Web of Science, Scopus, PubMed, IEEE Xplore, and Engineering Village-Compendex) from February 2024 to April 2025. The keyword strategy combined terms related to TAVI and computational techniques (e.g. CFD, FEA, and FSI) without restricting to durability-specific terms (see Supplementary material online, *Appendix*). This broad approach was chosen after preliminary testing showed that using narrower keywords (e.g. SVD, degeneration, thrombosis) missed many relevant studies, particularly engineering papers in which durability mechanisms were explored indirectly through simulations. Only peer-reviewed articles in English were included, with no restrictions on publication year. Reviews, book chapters, conference abstracts, and pre-print papers were excluded. The search was completed on the 4th April 2025.

### Data analysis

Two reviewers, one with a clinical background (E.R.) and the other with expertise in bioengineering and fluid mechanics (L.B.), independently conducted the search and screened titles and abstracts to exclude irrelevant studies (e.g. non-TAVI-specific, not using computational modelling, or focused solely on technical aspects without clinical relevance). They then reviewed and compared their selections, agreeing on the records to be assessed at a full-text level. Full texts of eligible articles were reviewed jointly, with disagreements resolved through discussion and, if needed, input from a third reviewer (C.H.R.).

As the review focused on SVD and leaflet thrombosis mechanisms, studies that primarily addressed procedural complications (e.g. PVL, coronary obstruction, aortic complications) or utilized modelling only for device performance or procedural planning were excluded unless they specifically investigated mechanisms related to valve degeneration.

Given the heterogeneity in modelling methodologies and study endpoints, no meta-analysis was conducted. Instead, we adopted a structured narrative approach to evaluate computational strategies and metrics used across the studies, their relevance for understanding valve degeneration mechanisms, and potential clinical implications. Progress towards verification, validation, and uncertainty quantification was also examined to assess the current state of computational modelling for realistic use in clinical research and practice.

## Results

**Figure 1:**
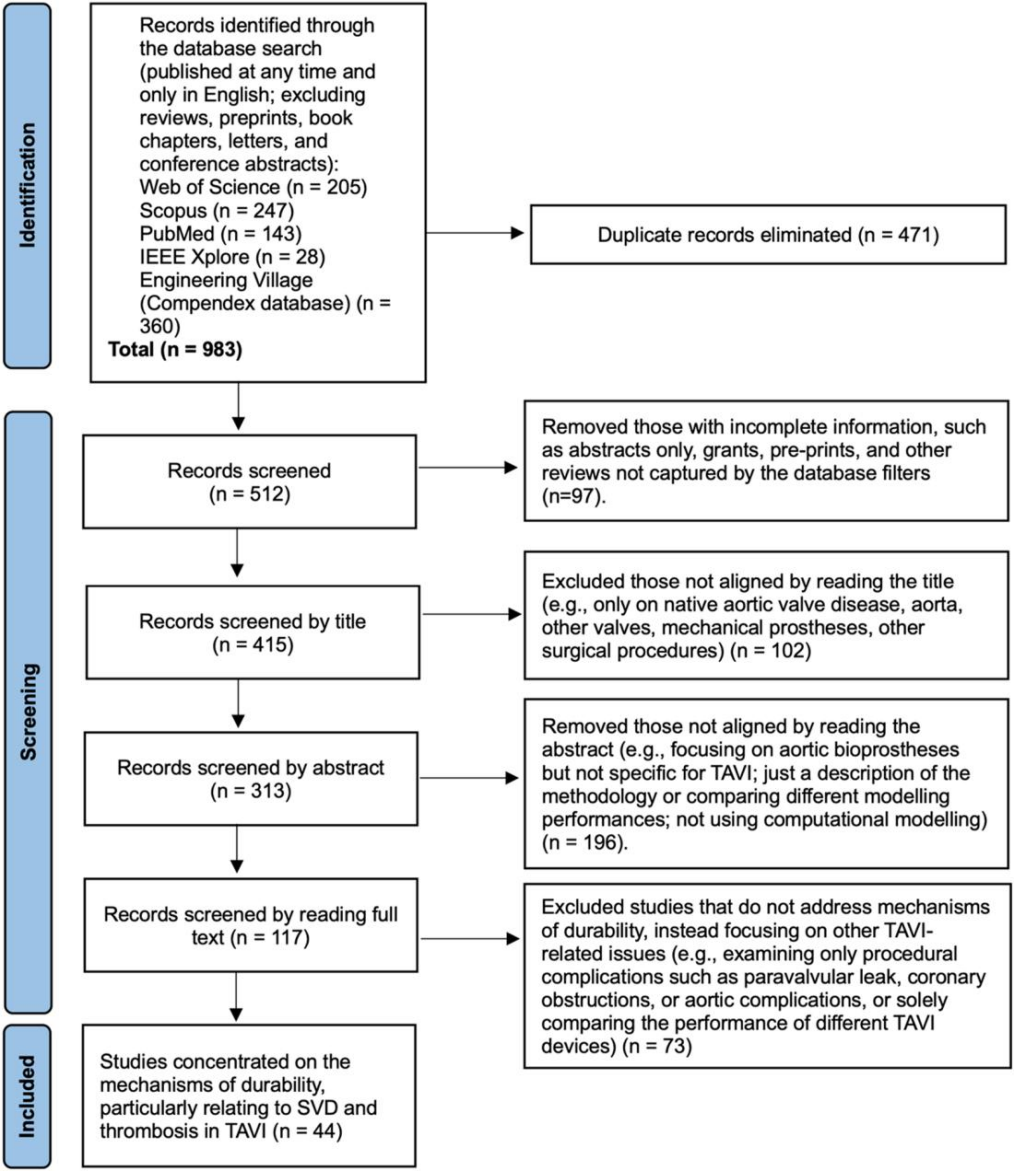
Study selection according to PRISMA guidelines. *From:* Page MJ, McKenzie JE, Bossuyt PM, Boutron I, Hoffmann TC, Mulrow CD, *et al*. The PRISMA 2020 statement: an updated guideline for reporting systematic reviews. BMJ 2021;372:n71. doi: 10.1136/bmj.n71.

*Figure 1* summarizes the study selection process following Preferred Reporting Items for Systematic Reviews and Meta-Analyses guidelines. A total of 983 records were initially identified. After removing 471 duplicates, 512 articles were screened by title and abstract. Following this, 117 full-text articles were assessed for eligibility by both researchers. Studies that primarily investigated procedural complications without direct relevance to long-term valve degeneration were excluded. Forty-four papers were ultimately included as directly addressing durability-related mechanisms: 13 focused on SVD mechanisms, 20 on TAV thrombosis, and 11 on design-related factors influencing TAV durability.

Most studies on SVD and device design applied FEA to simulate TAV implantation and assess stress distributions within the device and surrounding anatomy, aiming to evaluate potential mechanisms of mechanical failure. Many incorporated FSI to account for dynamic interaction between blood flow, the prosthesis, and native structures, providing insights into mechanical behaviours and leaflet fatigue over time. TAV thrombosis studies used CFD and FSI to assess turbulence, flow stasis, and shear stresses around TAV devices to evaluate thrombogenicity. Although these metrics do not directly measure durability, they can inform mechanisms of SVD and TAV thrombosis, the main determinants of TAV durability. Only one study proposed a direct measure of durability.^21^

Some studies combined computational techniques with *in vitro* experiments to validate predicted flow conditions and structural stress estimates in experimental settings, highlighting the potential of modelling to investigate TAV thrombosis- and SVD-related mechanisms.^22–24^ However, few studies compared simulated metrics with real-world outcome data to confirm links with durability outcomes.^25–31^

## Discussion

Computational modelling has the potential to advance our understanding of TAV degeneration mechanisms. While many studies have used generalized *in silico* models to explore anatomical, haemodynamic, and procedural contributors to SVD and thrombosis, patient-specific simulations provide deeper insights that could support personalized treatment planning to minimize risks.^28,30,32–35^

Regarding the durability-related issue investigated, some studies have focused on device design factors potentially influencing long-term performance and deterioration, simulating mechanical behaviours under varying conditions to inform durability improvements. These are summarized in Supplementary Discussion and Supplementary material online, *Table S1*. The discussion below focuses on studies exploring SVD and TAV thrombosis mechanisms, given their stronger clinical relevance. *Tables 2* and *3* summarizes the main findings, while the discussion below highlights those with clearer clinical implications.

**Table 2:** Mechanisms of SVD and mechanical failure in TAVI

Authors	Year	Computational methods	Title	Main findings
Crugnola L. *et al.*^25^	2025	CFD	Computational haemodynamic indices to identify Transcatheter Aortic Valve Implantation degeneration	Early post-TAVI disturbed flow, characterized by high Oscillatory Shear Index (OSI), low Time-Averaged Wall Shear Stress (TAWSS), and elevated flow reversal ratio measured on the leaflet surface (especially at the belly and free-edge regions) were associated with later SVD. A synthetic haemodynamic score combining these metrics effectively distinguished patients who developed SVD from those who did not.
Baylous K. *et al.*^36^	2024	FEA	In silico fatigue optimization of TAVR stent designs with physiological motion in a beating heart model	FEA-based TAVI stent fatigue model found higher strain values and risk of stent failure in stents with reduced strut width and better fatigue resistance in optimized designs, under physiologically representative loading conditions.
Fumagalli I. *et al.*^26^	2023	FSI	Fluid–structure interaction analysis of transcatheter aortic valve implantation	FSI simulations predict TAV degeneration based on pre-implantation data, correlating structural deterioration of TAV leaflets with proximal aortic wall shear stress distribution.
Bailoor S. *et al.*^37^	2022	FSI	Prosthetic Valve Monitoring via In Situ Pressure Sensors: In Silico Concept Evaluation using Supervised Learning	FSI with a data-driven analysis and ML finds wireless pressure sensors placed on the TAV stent can detect prosthetic valve dysfunction through reduced leaflet motion in a proof of concept sensorised TAVI system.
Bressloff N.W.^38^	2022	FEA	Leaflet Stresses During Full Device Simulation of Crimping to 6 mm in Transcatheter Aortic Valve Implantation, TAVI	Investigate the impact of crimping and leaflet material and thickness on the distribution and magnitude of stresses in TAV leaflets, identifying regions vulnerable to structural failure and impacting TAVI durability.
Zhang W. *et al.*^39^	2021	FEA	Simulating the time evolving geometry, mechanical properties, and fibrous structure of bioprosthetic heart valve leaflets under cyclic loading	Predict complex shape and fibre orientation changes of valve leaflets due to cyclic loading, finding significantly stiffer leaflets after loading and the highest degrees of stretch and fibre realignment in the belly and free-edge region of the leaflets.
Xiong T-Y. *et al.* ^35^	2021	FEA	Force distribution within the frame of self-expanding transcatheter aortic valve: Insights from in-vivo finite element analysis	Analyse amounts and distributions of forces within a TAV stent, imposed by the anatomy, with most of the force exerted at the level of the nadir, higher than native annulus, and unevenly distributed along the cross-sectional axis.
Johnson E.L. *et al.*^40^	2020	FSI	Thinner biological tissues induce leaflet flutter in aortic heart valve replacements	Blood flow disturbances and leaflet irregularities induced by thinner TAV leaflets and link with cardiovascular dysfunction and reduced valve durability.
Bailey J. *et al.*^33^	2017	FEA	The impact of imperfect frame deployment and rotational orientation on stress within the prosthetic leaflets during transcatheter aortic valve implantation	Imperfect TAV frame deployment and orientation due to calcified native valve increases stress distribution in valve leaflets, leading to premature device failure.
Wu W. *et al.*^34^	2016	FSI, *in vitro* experiments	Fluid–Structure Interaction Model of a Percutaneous Aortic Valve: Comparison with an In Vitro Test and Feasibility Study in a Patient-Specific Case	FSI simulations closely matched *in vitro* tests in durability with a 0.42% difference in maximum leaflet opening. Aortic root anatomy significantly affects stent strain distribution, indicating that standard *in vitro* tests may not fully represent real valve behaviour.
Martin C. *et al.*^21^	2015	FEA	Comparison of transcatheter aortic valve and surgical bioprosthetic valve durability: A fatigue simulation study	Use FEA to compare TAV and SAV leaflet fatigue, finding higher stresses and reduced durability for TAV leaflets, estimated at about 7.8 years.
Sun W. *et al.*^41^	2010	FEA, CFD	Simulated elliptical bioprosthetic valve deformation: Implications for asymmetric transcatheter valve deployment	Elliptical TAVs, caused by implantation in calcified aortic roots, significantly increase leaflet stress by 143% and the likelihood of central leakage compared with symmetric configuration.
Dwyer H.A. *et al.*	2009	CFD	Computational fluid dynamics simulation of transcatheter aortic valve degeneration	TAV sclerosis (35% orifice reduction) increased total force on the TAV leaflets by 63%. TAV Stenosis (78% orifice reduction) led to an 86% increase in total force and significant changes in peak shear stress, especially at the leaflet tips during systolic flow.

See the list of abbreviations from *Table 1*.

**Table 3:** Mechanisms of TAV thrombosis

Authors	Year	Computational method	Title	Main findings
Oks D. *et al.*^32^	2024	FEA, FSI, platelet activation modelling	Effect of sinotubular junction size on TAVR leaflet thrombosis: a fluid–structure interaction analysis	Simulations of Medtronic Evolut TAV deployment in three aortic models with varying sinutubular junction (STJ) sizes (26, 30, 34 mm) showed that the 30 mm STJ provided the most favourable haemodynamics (low pressure gradient, reduced platelet stress). Smaller STJs impaired stent expansion and flow, affecting the performance; larger STJs improved washout but increased thrombogenic risk.
Bornemann K.M. *et al.*^42^	2024	FSI, platelet activation modelling	The relation between aortic morphology and transcatheter aortic heart valve thrombosis: Particle tracing and platelet activation in larger aortic roots with and without neo-sinus	FSI simulations of TAVI in two generic aortic root models—one with thrombosis and the other not, each with and without a neo-sinus—showed that larger aortic roots had reduced sinus washout and higher platelet activation indices, particularly in the neo-sinus. Particles stagnating in the neo-sinus had the highest thrombogenic potential.
Khodaei S. *et al.*^29^	2023	FSI	Early detection of risk of neo-sinus blood stasis post-transcatheter aortic valve replacement using personalized haemodynamic analysis	Find substantial risks of blood stagnation in the neo-sinus and insufficient coronary flow relief post-TAVI, regardless of clinical state.
Borowski F. *et al.*^43^	2023	FSI	In silico model to assess thrombosis risk of TAVR with haemodynamic predictors using fluid structure interaction	Use haemodynamic metrics to quantify platelet activation and aggregation and predict risk of SLT.
Jahren S. *et al.*^30^	2023	FSI	Altered blood flow due to larger aortic diameters in patients with transcatheter heart valve thrombosis	Larger aortic dimensions, particularly at the sino-tubular junction and ascending aorta, are linked to increased risk of thrombosis due to unfavourable hemodynamics.
Barrett A. *et al.*	2023	FSI	A model of fluid–structure and biochemical interactions for applications to subclinical leaflet thrombosis	Simulate mechanisms of subclinical leaflet thrombosis (SLT) using an FSI-based thrombosis model, to show changes in valve dynamics and provide insights into clot deposition mechanisms.
Kovarovic B.J. *et al.*^22^	2023	FSI, *in vitro* experiments	Mild Paravalvular Leak May Pose an Increased Thrombogenic Risk in Transcatheter Aortic Valve Replacement (TAVR) Patients-Insights from Patient-Specific In Vitro and In Silico Studies	Mild PVL increases thrombogenic risk due to patient-specific platelet trajectories.
Qiu D. *et al.*^44^	2022	CFD, FSI, *in vitro* experiments	Structural analysis of regional transcatheter aortic valve under-expansion and its implications for subclinical leaflet thrombosis	Regional stent under-expansion in 26 mm CoreValve TAV device impairs leaflet motion and increases thrombosis risk.
Oks D. *et al.*^45^	2022	FSI	Fluid–structure interaction analysis of eccentricity and leaflet rigidity on thrombosis biomarkers in bioprosthetic aortic valve replacements	Increased annulus eccentricity and leaflet rigidity in TAVI reduce valve performance and increase thrombogenic and calcification risks.
Borowski F. *et al.*^23^	2022	FSI, *in vitro* experiments	Validation of a Fluid Structure Interaction Model for TAVR using Particle Image Velocimetry	Validate a numerical FSI model against *in vitro* data to identify thrombosis risks in TAVI, confirming its potential for clinical application.
Nappi F. *et al.*^31^	2021	FEA	Corevalve vs. Sapien 3 transcatheter aortic valve replacement: A finite element analysis study	Residual calcifications cause valve distortion and increased stress on the inner aortic wall in contact with the stent, leading to haemodynamic disturbances and thrombosis risks in TAVI.
Hatoum H. *et al.*^27^	2021	CFD, scaling-based argument	Predictive Model for Thrombus Formation After Transcatheter Valve Replacement	Derive a vorticity flux using simple scaling arguments that can accurately predict leaflet thrombosis post-TAVI based on personalized anatomical and flow parameters.
Singh-Gryzbon S. *et al.*^28^	2020	CFD	Influence of Patient-Specific Characteristics on Transcatheter Heart Valve Neo-Sinus Flow: An In Silico Study	Unique post-TAVI geometries, significantly influenced by sinus diameter, correlate with thrombus formation due to distinct, patient-specific flow stasis patterns.
Stiehm M. *et al.*	2019	FSI, CFD	Computational flow analysis of the washout of an aortic valve by means of Eulerian transport equation	Use FSI and a Eulerian approach to evaluate blood residence times, demonstrating significantly higher residence times in the sinus compared with the main flow and the value of residence time to quantify poor washout and increased thrombogenic potential for TAV devices.
Kopanidis A. *et al.*^46^	2015	CFD	Aortic flow patterns after simulated implantation of transcatheter aortic valves	The Edwards SAPIEN and Medtronic CoreValve induced different aortic flow patterns, with the Medtronic valve showing higher aortic wall shear stress and persistent vortex formation, potentially increasing the risk of vascular remodelling and thrombosis.
Mayo RP. *et al.*	2021	CFD	Impact of BASILICA on the thrombogenicity potential of valve-in-valve implantations	CFD simulations shows that BASILICA technique reduces thrombosis risk in ViV procedures, with Lagrangian methods for evaluating flow residence times providing better predictions than Eulerian methods.
Mayo, RP. *et al.*^47^	2020	CFD	Numerical models for assessing the risk of leaflet thrombosis post-transcatheter aortic valve-in-valve implantation	Find higher leaflet thrombosis risk in Edwards SAPIEN 3 devices due to intra-annular implantation, with risk locations identified by different measures (Eularian vs. Lagrangian) near-wall stagnation.
Vahidkhah K. *et al.*^48^	2017	FSI	Blood Stasis on Transcatheter Valve Leaflets and Implications for Valve-in-Valve Leaflet Thrombosis	Patient-specific FSI modelling showed that TAVs in ViV settings experience higher blood residence time on TAV leaflets, increasing thrombosis risk compared with SAVs.
Vahidkhah K. *et al.*^49^	2017	FSI	Supra-annular Valve-in-Valve implantation reduces blood stasis on the transcatheter aortic valve leaflets	Supra-annular TAV positioning in ViV settings reduces blood residence time on TAV leaflets and thrombosis risk, compared with intra-annular positioning.
Vahidkhah K. *et al.*^24^	2017	FSI, *in vitro* experiments	Valve thrombosis following transcatheter aortic valve replacement: significance of blood stasis on the leaflets	The geometric confinement in TAV/ViV procedures increases blood residence time on TAV leaflets, raising thrombosis risk compared with SAVR.

See the list of abbreviations from *Table 1*.

### SVD and mechanical failure in TAVI

Unlike SAVR, where the native valve is removed, TAVI deploys a prosthetic valve mounted on a rigid frame within the calcified native valve, creating unique haemodynamic conditions that may promote SVD. These include increased turbulence in the aortic root, and elevated mechanical stress on the leaflets and frame. Computational modelling, especially CFD and FSI, enables in-depth analysis of these metrics, allowing estimation of likely SVD and long-term mechanical failure. Crugnola *et al*.^25^ used patient-specific CFD simulations based on post-TAVI CT and echocardiographic data to evaluate flow across the cardiac cycle, applying patient-specific boundary conditions, and detailed TAV leaflet geometry. Disturbed flow patterns, characterized by local reverse flow index, high oscillatory shear index, and low time-averaged wall shear stress (WSS) values, indicating prolonged low magnitude stresses on the belly and free-edge regions of the leaflets, were associated with later SVD. Based on these metrics, they proposed a composite synthetic score that successfully distinguished patients with and without SVD. Further durability-linked metrics, not intuitively associated with degeneration mechanisms, can be identified from modelling studies. For instance, Fumagalli *et al*.^26^ used patient-specific FSI simulations based on pre-TAVI anatomy to estimate post-implantation haemodynamics. They found that persistently elevated WSS on the proximal aortic wall during early systole correlated with leaflet degeneration more reliably than pressure gradients. Together, these studies highlight the potential prognostic value of early haemodynamic patterns in predicting long-term SVD risk, and show how modelling could complement echocardiography by detecting flow abnormalities not visible on conventional imaging, potentially enhancing risk stratification and follow-up (*Table 2*).

Simulations based on FEA can clarify how TAVI-specific factors, such as crimping, asymmetric expansion, and deployment issues, contribute to SVD by increasing mechanical stress on the leaflets and frame,^8,50^ amplifying turbulence, and promoting degenerative processes.^51^ Crimping, for instance, can cause leaflet thinning, leading to damage and wear, accelerating calcifications, and thrombosis.^50^ A FEA study has shown that leaflet stress peaks near suture lines during crimping, and this can be reduced using thicker or polymeric leaflets, emphasizing the importance of leaflet material properties in TAVI durability.^33,38,41^ Incomplete frame expansion, often due to anatomical variations or residual native annular calcifications, can cause uneven force distribution, particularly at the commissures and stent attachments, increasing leaflet stress, and accelerating SVD.^33,41^ These findings highlight the potential of modelling to optimize valve selection and deployment strategies in anatomically complex cases, to preserve leaflet integrity and long-term durability.

Mechanical fatigue, distinct from calcific degeneration, is another contributor to SVD. It results from mechanical stress from repetitive cardiac cycles, leading to microdamage, especially in non-calcified high-stress regions, even when the tissue remains biologically intact.^8,50^ Fatigue affects both leaflets and frames. While traditionally assessed *in vitro* through accelerated wear testing, patient-specific simulations offer more realistic estimations.^34^ Martin *et al*.^21^ used FEA with a model developed to describe leaflet fatigue damage to simulate cyclic device loading. Higher stresses were observed in TAV leaflets, allowing estimation of reduced durability (∼7.8 years) compared with SAVR illustrating the potential of modelling for predicting durability timelines. However, the study considered only fatigue-based leaflet failure, used idealized geometries with material properties calibrated to SAV experiments, and could not simulate TAV to failure because of numerical instability, requiring extrapolation from the last simulated cycle. Fatigue modelling also helps identify stress-prone regions to inform design optimization.^,35,36,39^ These insights, often unattainable through experiments, underscore the value of simulation in evaluating device longevity under real-world conditions, and in refining implantation techniques and prosthesis designs to minimize fatigue-related deterioration. However, further modelling advances and experimental and validation studies are needed to aid calibration and confirm findings.

### TAV thrombosis

TAV thrombosis, particularly SLT, is an important cause of haemodynamic valve deterioration and poses unique challenges for TAVI durability.^15^ SLT is often asymptomatic and detectable as hypo-attenuated leaflet thickening (HALT) with/without reduced leaflet mobility on MDCT, can appear within 30 days post-procedure, and is more prevalent in TAVI (12–38%) than SAVR.^52^ While it may resolve spontaneously, SLT is linked with increased transvalvular gradients and possible progression to SVD.^7,52,53^ However, the unclear mechanisms and management highlight the need to better understand its role in TAVI longevity (*Table 3*).

The pathophysiology of TAV thrombosis reflects complex interactions between host (e.g. comorbidities, anatomy) and device-related factors (e.g. valve design, implantation techniques) unique in TAVI. Implanting a TAV within a calcified native valve creates two regions: the reduced native sinus (between displaced native leaflets and the aortic wall), and the neo-sinus (between TAV leaflets and native leaflets/stent complex).^52,54^ The neo-sinus is prone to thrombus due to increased blood stasis, platelet aggregation on biomaterial, and high shear stresses, causing focal vascular trauma, all of which are known to contribute to and promote thrombus formation. The retained native valve also acts as a pro-thrombotic stimulus.^52^ These phenomena are intensified in valve-in-valve (ViV) implantation due to the geometric confinement of TAV by the failed bioprosthesis, and are more pronounced in intra-annular vs. supra-annular positioning.^24,47–49^

While the complexity of TAV thrombosis makes direct prediction difficult, computational modelling can clarify the factors associated with its initiation and progression, and their links with SVD. This includes shear stresses within the blood that can cause platelet activation; low oscillatory wall shear stresses at the aortic wall that can create a pro-thrombotic environment via endothelial injury; and blood stasis that promotes accumulation and aggregation of activated platelets. These insights may support risk stratification, strategies to reduce thrombosis, and refinement of device design to improve durability.

CFD and FSI analyses can help elucidate the unique haemodynamic environment around TAVI devices. By tracking particles within the flow, platelet activation risk can be quantified by calculating the cumulative stresses they experience over time. Bornemann *et al*.^42^ applied this approach along with high-fidelity FSI to evaluate thrombosis risk in two generic aortic root models: a larger one representing the TAV thrombosis model, with and without the neo-sinus, and a smaller one representing unaffected TAVI patients. They found poorer sinus washout and the highest platelet aggregation indices in stagnating particles in the larger aorta, particularly when the neo-sinus was present. Although this could explain the higher rates of thrombosis induced in TAVI vs. SAVR, which lacks a neo-sinus, they only considered two generic aortic models with a simplified neo-sinus geometry. Additionally, only a single systolic acceleration was simulated because of computational cost, and diastolic flow stagnation was not fully explored. Oks *et al*.^32^ used similar methods, using particle residence time (indicating time spent within a volume) as an additional measure of thrombosis risk in a modified patient-specific anatomy with different sinotubular junction sizes. The smallest size showed the highest thrombotic risk and the poorest pressure gradients and TAV expansion, while the largest behaved similarly to the intermediate size but with slightly increased thrombosis risk. Although only one patient-specific case was considered and its anatomy modified, preventing comparison with clinical outcome data, such work helps identify anatomical drivers of thrombosis when clinical data are sparse. The potential of alternative metrics, such as flow circulation in the neo-sinus derived from scaling arguments, to predict leaflet thrombus occurrence was shown by Hatoum *et al*.^27^ By comparing this empirically-derived metric to post-TAVI CT thrombus volumes, they found it predicted outcomes better than usual CFD metrics like flow stasis and WSS. They note the small six-patient study requires a prospective study and more suitable FSI methods for improved measures.

Patient-specific models comparing simulated flow characteristics and risk metrics with observed TAV thrombosis have provided further insights into how anatomical variations may influence device hemodynamics and thrombosis risk.^28–31^ Features such as aortic root size,^30^ calcification distribution,^31^ post-TAVI aortic geometry,^28^ and systemic flow conditions^29^ can significantly affect sinus and neo-sinus washout and long-term outcomes. Although these studies are based on small cohorts (4–26 cases) and need validation with larger populations and outcome data to confirm their findings, these insights can help identify anatomies prone to poor washout, such as large or asymmetric roots, and support tailored procedural planning, including valve type selection and implantation strategy. This could promote a more personalized approach to risk stratification and may guide targeted post-procedural management, including pharmacological therapy where appropriate.

Further studies have shown that deployment,^44,45^ and device design^46^ also influence thrombogenicity. FSI and FEA studies found that regional frame under-expansion,^44^ and annulus eccentricity^45^ can impair leaflet motion, increasing blood stasis and mechanical stress on the leaflets, thus raising thrombotic and SVD risk. Although these studies use idealized geometries, neglecting patient-specific factors that could influence dynamics, they replicate experimental conditions used during regulatory testing, providing potential insights to optimize device design and approval.

Computational modelling can also clarify how commonly acceptable TAVI complications, such as mild PVL, may contribute to thrombogenicity. FSI simulations of patient-specific 3D printed TAVI replicas showed that platelet trajectories vary by PVL channel shape, linking complex channel morphologies and platelet stress accumulations near regurgitant jets to increased thrombogenic risk.^22^ This level of insight, unattainable from echocardiography, underscores modelling’s potential for individual risk stratification and follow-up.

### Strengths and limitations

The main strength of this review is its multidisciplinary perspective, combining clinical and engineering expertise to evaluate the diverse modelling strategies and durability-related surrogate metrics used in TAVI, together with their potential clinical implications. Although a broad search was conducted, some engineering studies with less clinically oriented titles/abstracts may have been missed. Nevertheless, the included literature is consistent with prior reviews.^10–12^

Computational modelling cannot yet reproduce full cardiovascular complexity, so direct durability prediction is not feasible. However, short-term surrogate metrics, such as accumulated leaflet or stent mechanical stresses and indices of blood flow stasis, can offer mechanistic insights into pathways involved in SVD and thrombosis. The potential of a range of computational methods and durability-associated metrics, as well as additional metrics not intuitively associated with valve durability, has been shown in several small-scale studies, demonstrating potential links between simulated metrics and real-world durability outcomes.^25–27^ Simulations over short or specific time periods show potential in predicting long-term outcomes, despite not directly simulating full long-term scenarios.^21^ However, the range of modelling approaches and metrics is wide, no single method emerges as preferable across studies, and clinical relevance is often insufficiently specified.

Different computational methods used to calculate individual metrics will influence results due to assumptions within the models, as well as other factors such as patient- and device-specificity. Many computational studies used simplified geometries, lacked validated tissue and device material properties, relied on small case numbers, or explored limited flow scenarios, often restricted to pre-TAVI anatomical information from MSCT scans. This may cause models to overlook patient variability and post-implantation changes, potentially impacting device longevity. Factors such as pulsatile simulations being run long enough to sufficiently represent quasi-steady flow observed in physiological conditions, must also be considered.

Across the studies, a comprehensive verification, validation, and uncertainty quantification (VVUQ) pipeline, following the risk-informed credibility guidance for medical-device modelling, is largely missing, limiting confidence in accuracy and clinical relevance.^55,56^Verification checks the numerical correctness (e.g. mesh/time-step convergence, solver checks, and benchmarks). Validation tests model agreement with reality at two levels: (ⅰ) physical-field validation, comparing simulated pressure/velocity or leaflet/stent stress against benchtop or imaging-derived fields with quantitative error reporting; and (ⅱ) clinical outcome-level, where model-derived surrogates, after any necessary calibration to data, are assessed for association with, and ability to predict clinical endpoints (e.g. SVD, thrombosis, reintervention) using pre-specified cut-points or models. Uncertainty/sensitivity analyses quantify how modelling assumptions and input variability affect results, and thus reliability.^57^ Best practice for believable modelling results is to declare context-of-use (mechanistic insight vs. patient-level prediction) and credibility goals *a priori*, then report VVUQ per ASME V&V 40 standards, using independent datasets for outcome-level validation, applicability check, and documented uncertainty analyses.^55–57^

Most studies reported some verification and/or physical-field validation; far fewer connected simulations to limited clinical data. None completed a pre-specified, calibrated surrogate-to-outcome validation with formal uncertainty analysis. Approaches varied. Several FEA/FSI/CFD papers checked numerics or fields against benchtop data: simulated stent deformation, leaflet motion, or local flow vs. in-vitro measurements.^23,24,34,36,40,44^ Others performed imaging-based field checks, e.g. qualitative comparisons of simulated post-TAVI stent geometry with CT or matching Doppler/echo flow or effective orifice area where available.^25,26,28^ A few relied on solvers validated elsewhere without documenting validation for the present setup.^29,42,43,47^ Some provided partial validation only; for example, Martin *et al*.^21^ reported bench validation for a surgical valve model, but no equivalent TAV bench validation. Some studies reproduced known benchmark behaviours qualitatively or quantitatively, although reporting of convergence analyses or error quantification was uncommon.^28,45^ Only a handful of studies related simulated metrics to imaging readouts or small patient cohorts: e.g. Crugnola (scores distinguishing SVD vs. non-SVD with echo flow checks), Fumagalli (degeneration vs. wall-shear distribution with CT geometry checks), Hatoum and Singh-Gryzbon (thrombus volume/neo-sinus flow), Nappi (device-failure case), and Khodaei *et al*. (flow metrics vs. thrombosis).^25–29,31^ Importantly, none executed a pre-specified, calibrated surrogate-to-clinical-outcome validation with formal uncertainty analysis capable of supporting prediction of SVD, SLT, or reintervention. As it stands, modelling is best placed for hypothesis generation, mechanism exploration, and trial design, pending harmonized outputs, transparent reporting, and larger clinically linked datasets.

Finally, patient-specific datasets for model validation remain scarce, likely reflecting the challenge of consistently diagnosing SVD or SLT, and the older age of most TAVI populations, which limits longitudinal imaging and follow-up. Wider, standardized clinical/imaging protocols for detecting durability phenomena, and access to more diverse (including younger) TAVI populations, would provide better-annotated cases and enable robust patient-specific validation against durability outcomes.

### Clinical implications, open issues, and future directions

Durability remains a key concern in younger TAVI patients, whose longer life expectancy increases the likelihood of reinterventions, and calls for a lifetime rather than a single-procedure perspective.^58^ Younger patients are likely to require one or more subsequent interventions (TAVI-in-TAVI, TAVI-in-SAVR, or surgery after TAVI) once the index valve fails. In this context, computational modelling can inform the entire valve-replacement pathway. Pre-procedurally, patient-specific simulations can evaluate device type, implantation depth, and commissural alignment to preserve coronary access and annular geometry, facilitating future valve-in-valve procedures. In the reintervention setting, modelling can help anticipate transvalvular gradients, coronary obstruction risk, and residual neo-sinus blood stasis to assess the feasibility of repeat TAVI vs. surgery. Embedding these simulations into the decision pathway has the potential to shift their role from purely mechanistic exploration to tools supporting lifetime planning and shared decision-making.

Although short- to mid-term clinical trials and registries suggest satisfactory TAVI performance and no clear inferiority to SAVR in older high-risk populations, data beyond 10 years are scarce and younger or anatomically challenging groups, such as those with bicuspid aortic valves, ViV procedures, and complex anatomies, remain largely underrepresented.^59^ The true assessment of TAVI durability can only come from extended clinical follow-up, but these data will take years to accumulate. In the meantime, modelling can provide mechanistic insights into factors influencing device durability, such as leaflet stress, stent deformation, and flow abnormalities. By linking simulated metrics to known outcomes from existing clinical datasets, modelling can help identify which mechanical and haemodynamic conditions are most associated with SVD or thrombosis risk, supporting hypothesis generation and prioritization of metrics for early detection of degeneration in future clinical trials.

Modelling can also reveal phenomena that conventional imaging and experimental testing cannot fully capture. For instance, CFD simulations have shown that subtle stent asymmetry or ‘pinwheeling’ generates turbulent flow and abnormal shear stresses, conditions believed to accelerate leaflet wear and thrombosis.^34,60^ When integrated with imaging datasets, such models may help refine the interpretation of subtle flow abnormalities, and suggest conditions that could predispose to early dysfunction, providing a framework for future preventive strategies aimed at improving long-term valve performance.

Despite the promises, clinical integration of modelling still faces major challenges. Most current models target specific mechanisms (fatigue, thrombosis, and calcification), use limited patient-specific detail, and rely on surrogate markers rather than direct predictors of valve lifespan. Although bench/imaging checks build credibility for the physics, predicting durability from surrogates needs two extra steps: first, calibrate the surrogate (e.g. shear/platelet activation indices, fatigue indices) to real data (e.g. fitting a platelet-activation constant or an S–N fatigue curve); and second, test the calibrated metric prospectively, with pre-specified criteria, against clinical endpoints (SVD, leaflet thrombosis, reintervention) in an independent cohort (ideally external) with strict separation from calibration, blinding where feasible, uncertainty analysis, and transparent performance reporting.^55,56^This outcome-level step is rare in current durability modelling, which limits credible prediction and, in turn, clinical applicability. Additonally, important pathophysiological factors contributing to durability, such as comorbidities, inflammation, calcification dynamics, and biological responses, remain difficult to represent numerically, limiting the physiological realism of current models. Without these elements, models provide valuable but partial representations of the *in vivo* environment. Close collaboration between engineers and clinicians is needed so modelling choices address clinically meaningful questions and results are interpreted appropriately.

Finally, clinical translation is also constrained by practical barriers, including computational demands, workflow complexity, data governance, and training. Runtime and operational complexity can be reduced by utilizing advanced methods and machine learning (ML) techniques to provide practical solutions on realistic clinical timescales. Progress also requires secure data pipelines that support VVUQ reporting, ML-based calibration, and, where feasible, embedded sensors providing real-world signals.^37,61,62^ Ultimately, an interdisciplinary ecosystem linking engineers, clinicians, and data scientists is needed to overcome these barriers and enable sustainable integration of modelling into the TAVI pathway.^63^

## Conclusions

Computational modelling provides mechanistic insights into TAVI deterioration processes, including SVD and TAV thrombosis, which can help inform design and procedural improvements. However, these models currently do not directly measure durability; they typically simulate implantation or short-term haemodynamics and provide surrogate metrics (e.g. leaflet stress, stent deformation, flow stasis) that are plausibly linked to degeneration but are not validated predictors of long-term valve lifespan. Accordingly, models should be viewed as complementary, hypothesis-generating tools alongside clinical and experimental data, not as standalone durability assessors.

To move toward credible clinical use, models need transparent verification, rigorous two-level validation, including calibration and validation of surrogate metrics against clinical data, and routine uncertainty/sensitivity reporting. With larger longitudinal datasets and rigorous VVUQ, modelling can take a meaningful role in lifetime planning for younger patients more likely to require reintervention. Delivering this will require tight collaboration between engineers, clinicians, and data scientists to advance methods with realistic clinical relevance for TAVI durability.

## Supplementary Material

ztag020_Supplementary_Data

## Data Availability

No new data were generated or analysed in support of this research.
