# Supplementary material for: Understanding the mechanisms of TAVI durability through computational modelling: a multidisciplinary review
Source: Eur Heart J Digit Health. 2026 Feb 3;7(2):ztag020. doi: 10.1093/ehjdh/ztag020 (PMC12912916; doi:10.1093/ehjdh/ztag020)
Supplement: ztag020_Supplementary_Data [file ztag020_supplementary_data.pdf]

Supplementary Materials

1. **Search keywords across the databases**
2. **Supplemental discussion. Optimising TAVs design and durability**
3. **Supplemental Table 1. Improving TAVs design and durability through computational modelling**
4. **Supplemental references.**

1. Search keywords across the databases

Web of Science

(AB=(computational fluid dynamics) OR AB=(CFD) OR AB =(fluid-structure interaction) OR AB=(FSI) OR AB =(computational simulations and analysis) OR AB =(computational modelling and analysis))

AND

(AB=(bioprosthetic aortic valve) OR AB=(aortic valve prosthesis) OR AB=(aortic valve replacement) OR AB=(bioprosthesis) OR AB=(bioprosthetic) OR AB=(transcatheter aortic valve replacement) OR AB=(TAVR) OR AB=(transcatheter aortic valve implantation) OR AB=(TAVI) OR AB=(aortic valve prosthesis implantation))

==

Scopus

(ABS (computational fluid dynamics) OR ABS (CFD) OR ABS (fluid-structure interaction) OR ABS (FSI) OR ABS(computational simulations and analysis) OR ABS(computational modelling and analysis))

AND

(ABS(bioprosthetic aortic valve) OR ABS(aortic valve prosthesis) OR ABS(aortic valve replacement) OR ABS(bioprosthesis) OR ABS(bioprosthetic) OR ABS(transcatheter aortic valve replacement) OR ABS(TAVR) OR ABS(transcatheter aortic valve implantation) OR ABS(TAVI) OR ABS(aortic valve prosthesis implantation))

==

PubMed

((computational fluid dynamics [Title/Abstract]) OR (CFD[Title/Abstract]) OR (fluid-structure interaction [Title/Abstract]) OR (FSI [Title/Abstract]) OR (computational simulations and analysis [Title/Abstract]) OR (computational modelling and analysis [Title/Abstract]))

AND

((bioprosthetic aortic valve [Title/Abstract]) OR (aortic valve prosthesis [Title/Abstract]) OR (aortic valve replacement [Title/Abstract]) OR (bioprosthesis [Title/Abstract]) OR (bioprosthetic [Title/Abstract]) OR (transcatheter aortic valve replacement [Title/Abstract]) OR (TAVR [Title/Abstract]) OR (transcatheter aortic valve implantation [Title/Abstract]) OR (TAVI [Title/Abstract]) OR (aortic valve prosthesis implantation [Title/Abstract]))

==

IEEE Xplore

(("All Metadata": computational fluid dynamics) OR ("All Metadata": CFD) OR ("All Metadata": fluid-structure interaction) OR ("All Metadata": FSI) OR ("All Metadata": computational simulations and analysis) OR ("All Metadata": computational modelling and analysis))

AND

(("All Metadata": bioprosthetic aortic valve) OR ("All Metadata": aortic valve prosthesis) OR ("All Metadata": aortic valve replacement) OR ("All Metadata": bioprosthesis) OR ("All Metadata": bioprosthetic) OR ("All Metadata": transcatheter aortic valve replacement) OR ("All Metadata": TAVR)) OR ("All Metadata": transcatheter aortic valve implantation) OR ("All Metadata": TAVI) OR ("All Metadata": aortic valve prosthesis implantation))

==

Engineering Village - Compendex

((computational fluid dynamics) OR (CFD) OR (fluid-structure interaction) OR (FSI) OR (computational simulation and analysis) OR (computational modelling and analysis))

AND

((aortic valve prosthesis) OR (aortic valve replacement) OR (bioprosthesis) OR (bioprosthetic) OR (transcatheter aortic valve replacement) OR (TAVR) OR (transcatheter valve insertion) OR (TAVI) OR (aortic valve prosthesis implantation))

2. Optimising TAVs design and durability

Beyond exploring mechanisms of TAVI durability, computational modelling supports TAV design by enabling the development of more durable devices (Supplementary Table 1). By simulating devices *in silico* and adjusting variables like flow conditions, material properties, and design features, these models assess performance and durability across broader conditions than *in vitro* methods. This approach identifies and optimises key factors influencing mechanical performance, complications, and long-term durability, guiding the creation of robust, long-lasting devices while minimising procedural risks.

For instance, Barati et al.[1] used patient-specific geometry and FEA to optimise TAVI stent designs, improving crimping strain and stent eccentricity and enhancing device integrity and longevity. Liu et al.[2] and Van Aswegen et al.[3] examined stent designs and leaflet attachment configurations to identify options with more balanced force distributions throughout the device and reduced stress-prone regions, minimising mechanical fatigue and thrombosis. Ghosh et al.[4] applied FSI to investigate polymeric TAV leaflet designs, which hold promise for greater durability compared to traditional materials, identifying mechanical failure areas, and suggesting design improvements. Integrating ML with computational modelling can accelerate design optimisation, such as estimating deformed leaflet geometries and stress distributions within seconds. [5]

Although computational modelling provides unique insights unavailable through *in vitro* experiments, accurately selecting material properties and modelling methods is essential for capturing the complex dynamics of TAVI durability. For instance, Murdock et al.[6] highlighted the importance of accurate TAV leaflet material properties, while others showed the advantage of FSI over FEA, [7–10] for capturing leaflet dynamics and stress distributions

and identifying potential failure points. FSI revealed how haemodynamic factors, like fluid inertia through the valve, can influence leaflet deformation, providing key insights to enhance durability.[7,8]

Though most studies rely on *in silico* models, they offer predictive insights to guide the development of next-generation TAVI devices with improved durability, ultimately enhancing patient outcomes.

Supplemental Table 1. Improving TAVs design and durability through computational modelling

Authors	Year	Computational Method	Title	Main findings
TAV stent/frame and leaflet design				
Fries ER. *et al*	2023	FEA	Structural study of a polymeric aortic valve prosthesis. Analysis for a hyperelastic material.	Use FEA to simulate a polymeric aortic valve prosthesis, made with hyperelastic material, focusing on design optimization. Adding rounded fillets at leaflet-pillar junctions reduces maximum Von Mises stress by 33.9%, enhancing mechanical performance. Additional modifications, like adjusting leaflet thickness and inter-commissural distances, further improve TAV stress distribution under load.
Barati S. *et al*	2022	FEA	Patient-specific multi-scale design optimization of transcatheter aortic valve stents	Optimize TAV stent design using patient-specific geometry and FEA, achieving significant improvements in crimping strain, radial strength, stent eccentricity, and anchorage area, enhancing long-term device performance.

Liu X. *et al*	2022	FSI, CFD	Fluid-Structure Interaction Analysis on the Influence of the Aortic Valve Stent Leaflet Structure in Hemodynamics	Compare valve opening shape, orifice area, and stress-strain, haemodynamic flow and pressure distributions for three personalised stent system designs, to investigate enhanced stent design reliability and reduce thrombosis risk.
Liang L. *et al*	2019	ML (trained using FEA simulation outputs)	A proof-of-concept study of using machine-learning in artificial aortic valve design: From leaflet design to stress analysis	Use ML models trained on FEA outputs, in place of FEA to accurately estimate the stress and deformation of TAV leaflets from a given set of leaflet design parameters.
Ghosh RP. *et al*	2018	FSI	Comparative fluid-structure interaction analysis of polymeric transcatheter and surgical aortic valves' hemodynamics and structural mechanics	Develop polymeric TAV prototype and use FSI to show improved haemodynamic performance and mechanical leaflet stresses to the SAVR-specific version of the design, previously optimised to reduce leaflet stresses and thrombogenic potential.
Van Aswegen K.H.J. *et al*	2012	FEA, FSI	Investigation of leaflet geometry in a percutaneous aortic valve with the use of fluid-structure interaction simulation	Investigate impacts of different configurations of the leaflet's attachment to the surrounding stent on valve tissue fatigue.
Importance of parameters and methods				
Brown JA et a.	2023	FSI (using FE)	Patient–Specific Immersed Finite Element–Difference Model of Transcatheter Aortic Valve Replacement	Patient-specific FSI model to simulate realistic deployment and function of the CoreValve Evolut

				R, capturing detailed device-tissue interactions, stress distribution, and leaflet dynamics. Identify high-stress regions and potential failure points, offering insights to improve device design for durability and to mitigate other procedural complications like malpositioning and improper sealing.
Borowski F. *et al*	2018	FEA, FSI	Fluid-structure interaction of heart valve dynamics in comparison to finite-element analysis	FEA predicts faster total valve opening compared to FSI by a factor of 5. This suggests that fluid inertia that surrounds the valve leaflets, that is neglected in FEA, significantly affects leaflet deformation.
Murdock. *et al*	2018	FEA, *in vitro* experiments	Characterization of mechanical properties of pericardium tissue using planar biaxial tension and flexural deformation	FEA simulations show the importance of both flexural and biaxial tensile data compared to just biaxial for accurate mechanical property modelling and bioprosthetic leaflet design.
Mao W. *et al*	2016	FEA, FSI	Fluid-Structure Interaction Study of Transcatheter Aortic Valve Dynamics Using Smoothed Particle Hydrodynamics	FSI models show more realistic leaflet deformation and higher peak stresses due to fluid inertia compared to FEA-only models, while tissue anisotropy had minor haemodynamic impact but could reduce peak leaflet stress if tissue

				stiffness was lower in the radial direction.
Luraghi G. *et al*	2017	FEA, FSI, *in vitro* experiments	Evaluation of an aortic valve prosthesis: Fluid-structure interaction or structural simulation?	FSI simulations proved more accurate in replicating valve-fluid interactions observed in vitro, providing more realistic valve behaviour and fluid dynamic results than FEA simulations.

CFD: Computational fluid dynamics; FEA: Finite element analysis; FSI: fluid-structure interaction; TAVI: Transcatheter Aortic Valve Implantation.

5. Supplemental references

1 Barati S, Fatouraee N, Nabaei M, *et al.* Patient-specific multi-scale design optimization of transcatheter aortic valve stents. *Comput Methods Programs Biomed* 2022; 221. DOI:10.1016/j.cmpb.2022.106912.

2 Liu X, Zhang W, Ye P, Luo Q, Chang Z. Fluid-Structure Interaction Analysis on the Influence of the Aortic Valve Stent Leaflet Structure in Hemodynamics. *Front Physiol* 2022; 13. DOI:10.3389/fphys.2022.904453.

3 Van Aswegen KHJ, Smuts AN, Scheffer C, Weich HSV, Doubell AF. Investigation of leaflet geometry in a percutaneous aortic valve with the use of fluid-structure interaction simulation. *J Mech Med Biol* 2012; 12. DOI:10.1142/S0219519411004538.

4 Ghosh RP, Marom G, Rotman OM, *et al.* Comparative fluid-structure interaction analysis of polymeric transcatheter and surgical aortic valves' hemodynamics and structural mechanics. *J Biomech Eng* 2018; 140. DOI:10.1115/1.4040600.

5 Liang L, Sun B. A proof of concept study of using machine-learning in artificial aortic valve design: From leaflet design to stress analysis. *Bioengineering* 2019; 6. DOI:10.3390/bioengineering6040104.

6 Murdock K, Martin C, Sun W. Characterization of mechanical properties of pericardium tissue using planar biaxial tension and flexural deformation. *J Mech Behav Biomed Mater* 2018; 77: 148–56.

7 Borowski F, Sämann M, Pfensig S, *et al.* Fluid-structure interaction of heart valve dynamics in comparison to finite-element analysis. In: Current Directions in Biomedical Engineering. Walter de Gruyter GmbH, 2018: 259–62.

8 Mao W, Li K, Sun W. Fluid–Structure Interaction Study of Transcatheter Aortic Valve Dynamics Using Smoothed Particle Hydrodynamics. *Cardiovasc Eng Technol* 2016; 7: 374–88.

9 Luraghi G, Wu W, De Gaetano F, *et al.* Evaluation of an aortic valve prosthesis: Fluid-structure interaction or structural simulation? *J Biomech* 2017; 58: 45–51.

10 Brown JA, Lee JH, Smith MA, *et al.* Patient–Specific Immersed Finite Element–Difference Model of Transcatheter Aortic Valve Replacement. *Ann Biomed Eng* 2023; 51: 103–16.
